# Real-world use of HIF-PH inhibitors in the Japan dialysis outcomes and practice patterns study (J-DOPPS, 2019–2022)

**DOI:** 10.1186/s12882-026-04831-2

**Published:** 2026-02-19

**Authors:** Masahiro Eriguchi, Kazuhiko Tsuruya, Hisashi Noma, Yoshihiro Onishi, Masami Inuzuka, Kenji Harada, Masaomi Nangaku

**Affiliations:** 1 Department of Nephrology, Nara Medical University, 840 Shijo–cho, Kashihara, Nara 634-8521 Japan; 2 Department of Data Science, The Institute of Statistical Mathematics, Tachikawa, Japan; 3 Institute for Health Outcomes and Process Evaluation Research (iHope International), Kyoto, Japan; 4 Patient Driven Academic League (PeDAL), Kyoto, Japan; 5 Medical Affairs Department, Kyowa Kirin Co.,Ltd, Tokyo, Japan; 6 Division of Nephrology and Endocrinology, The University of Tokyo Graduate School of Medicine, Tokyo, Japan

**Keywords:** Hypoxia-inducible factor prolyl hydroxylase inhibitors (HIF-PHIs), Anemia in chronic kidney disease, Maintenance hemodialysis patients, Erythropoiesis-stimulating agents (ESAs), C-reactive protein (CRP)

## Abstract

**Background:**

Hypoxia-inducible factor prolyl hydroxylase inhibitors (HIF-PHIs) are a novel therapeutic alternative to erythropoiesis-stimulating agents (ESAs) for anemia in chronic kidney disease. Evidence regarding their real-world effectiveness is still limited.

**Methods:**

We analyzed data from the Japanese Dialysis Outcomes and Practice Patterns Study (J-DOPPS, 2019–2022) to assess the real-world effectiveness of HIF-PHIs among maintenance hemodialysis patients. We compared patients who transitioned from ESAs to HIF-PHIs with those who continued ESAs, focusing on baseline characteristics and changes in anemia-related, iron metabolism, and inflammatory parameters.

**Results:**

Among 1433 patients on ESAs, only 56 (3.9%) were switched to HIF-PHIs. Compared with the ESA continuation group, patients in the HIF-PHI group had poorer nutritional status (albumin: 3.3 vs. 3.6 g/dL; cholesterol: 142 vs. 156 mg/dL; respectively) and greater ESA resistance (erythropoietin resistance index: 18.4 vs. 8.5 IU/kg/week/g/dL, respectively), despite higher ESA doses and iron prescription rates. After switching, a significant increase in hemoglobin was observed at 1 month (+0.44 g/dL; 95% confidence interval: 0.05–0.82; *p* = 0.025). Serum iron and total iron-binding capacity levels also increased, while transferrin saturation and ferritin remained unchanged. A subgroup analysis suggested greater hemoglobin response in patients with high C-reactive protein levels (p for interaction = 0.074), but no difference by erythropoietin resistance index (p for interaction = 0.372).

**Conclusions:**

In routine clinical practice, HIF-PHIs were selectively prescribed to patients with treatment-refractory anemia. Treatment increased hemoglobin levels, with a suggestive trend toward greater effect among those with inflammation, supporting the potential utility of HIF-PHIs in complex anemia management.

**Supplementary information:**

The online version contains supplementary material available at 10.1186/s12882-026-04831-2.

## Introduction

Anemia in chronic kidney disease (CKD) has long been treated primarily with erythropoiesis-stimulating agents (ESAs), which have substantially improved patients’ quality of life [1, 2]. However, anemia management remains inadequate, and moderate-to-severe anemia remains prevalent among CKD patients [3].

Epidemiological studies based on observational data have consistently demonstrated that anemia in both dialysis-dependent and non-dialysis-dependent CKD (DD-/NDD-CKD) populations is associated with higher mortality [4, 5], increased cardiovascular events [6], and faster progression of kidney disease [5]. In contrast, major clinical trials of ESA therapy have shown that normalization of hemoglobin (Hb) levels does not necessarily lead to improved clinical outcomes in CKD patients [1, 2, 7, 8]. The Normal Hematocrit Cardiac Trial involving hemodialysis (HD) patients was terminated early because of a trend toward increased mortality in the group targeted to achieve normal hematocrit levels [7]. In NDD-CKD patients, Hb normalization above 13 g/dL was associated with an increased risk of cardiovascular events in the CHOIR trial [8], a significantly higher initiation rate of kidney replacement therapy in the CREATE trial [1], and an increased incidence of stroke in the TREAT trial [2]. These adverse outcomes are thought to be related to ESA hyporesponsiveness and/or high-dose ESA usage [9–13].

Hypoxia-inducible factor prolyl hydroxylase inhibitors (HIF-PHIs) are a novel class of agents that stimulate endogenous erythropoietin production by activating HIF, which regulates erythropoietin gene expression [14, 15]. As an alternative to ESAs, HIF-PHIs have shown efficacy in correcting and maintaining Hb levels in multiple phase 3 clinical trials [16]. In addition to erythropoiesis, HIF activation may produce pleiotropic effects, including improved iron metabolism and potential anti-inflammatory actions [15], thereby expanding its therapeutic potential. However, concerns remain regarding the long-term safety of HIF-PHIs, including their effects on cardiovascular events, thromboembolism, and malignancies [16]. Moreover, while evidence from randomized controlled trials exists, real-world data on the use of HIF-PHIs and their impact on anemia, iron metabolism, and inflammation in CKD patients remain limited.

This study aimed to evaluate the real-world effectiveness of HIF-PHIs to treat anemia among maintenance dialysis patients, using data from the Japanese Dialysis Outcomes and Practice Patterns Study (J-DOPPS, 2019–2022). Specifically, we compared patients who switched from ESA therapy to HIF-PHIs with those who continued on ESA therapy, assessed baseline patient characteristics at the time of switching, and examined subsequent changes in anemia-related parameters, iron metabolism, and inflammatory markers to clarify treatment patterns in clinical practice.

## Materials and methods

### Study design and data source

J-DOPPS is a prospective cohort study performed in Japan as part of the worldwide Dialysis Outcomes and Practice Patterns Study (DOPPS) [17]. The study population consisted of adult hemodialysis outpatients randomly selected from representative samples drawn from hemodialysis facilities in Japan. This study used data from J-DOPPS Phase 7, which took place from 2019 to 2022 and included 1519 patients. During the observation period of J-DOPPS 7, a HIF-PHI was first launched in Japan (roxadustat, in November 2019).

We addressed two sub-studies related to the switch from ESA therapy to HIF-PHIs: (1) What were the characteristics of patients who switched from ESA therapy to HIF-PHIs? and (2) How did the status of anemia and iron metabolism change after this switch? This study was approved by the Nara Medical University Ethics Committee (Approval Number: 3849).

### Sub-study 1:characteristics of patients who switched to HIF-PHIs

Study subjects. Sub-study 1 compared the characteristics of patients who switched from ESA therapy to HIF-PHIs with those who continued ESA therapy. Among patients who received ESA prescriptions during the observation period, those who received HIF-PHI prescriptions no later than 3 months after the last ESA prescriptions comprised the HIF-PHI group (exposure group), while those who continued ESA therapy (did not switch to HIF-PHI) comprised the ESA continuation group (control group).

Variables. Demographic characteristics, clinical laboratory values, and medications related to anemia were recorded for both groups. For the HIF-PHI group, the most recent measurements before the first HIF-PHI prescription were used, and for the ESA continuation group, the earliest measurements obtained during ESA therapy within the observation period were used. Additionally, changes in blood Hb, transferrin saturation (TSAT), serum ferritin (ferritin), serum iron, and total iron binding capacity (TIBC) from November 2019, just before the initial release of HIF-PHIs, to December 2021 were recorded for both groups.

### Sub-study 2: changes in anemia status after switching to HIF-PHIs

Study subjects. In Sub-study 2, the analysis included patients from the HIF-PHI group in Sub-study 1 who had at least one measurement of the relevant markers available after the prescription of HIF-PHIs.

Variables. We analyzed changes in Hb, TSAT, ferritin, serum iron, and TIBC before and up to 12 months after switching to HIF-PHIs. Additionally, we evaluated the interactions between changes in Hb and C-reactive protein (CRP) or the erythropoietin resistance index (ERI).

### Statistical methods

Continuous variables were summarized as means and standard deviations or medians and interquartile ranges, while categorical variables were summarized as frequencies and proportions.

In Sub-study 1, the extent of differences in patient characteristics between the two groups was assessed using standardized differences. Welch’s t-tests and Mann-Whitney’s U-tests were also used for continuous variables, and chi-square tests were used for categorical variables.

In Sub-study 2, point estimates and 95% confidence intervals (CIs) for each variable were obtained for each month before and after switching to HIF-PHIs using the generalized estimation equation (GEE) Gaussian regressions. In this analysis, the month was modelled as a discrete explanatory variable, and the working correlation matrix was set to AR (1). If intermittently missing outcome data are involved, we assumed these data are unchanged from the nearest past data and imputed them for the longitudinal outcome data.

To evaluate the magnitude of change before and after the switch, we estimated the average difference and the 95% CI using an interrupted time series analysis [18]. We used the standard segmented regression models via GEE Gaussian regressions. This analysis included data from 6 months before the switch through 12 months after. Particularly, we assumed the time trends before and after the switch were constant respectively in each individual patient.

To assess the interactions, we divided the population into two groups based on the median values of CRP or ERI, and conducted the tests of differences of the regression functions in the segmented regression models between the two groups.

The *p*-values quoted are two-sided, and the significance level was set at 5%. Given that the detectability of interaction effects is relatively low compared to main effects, a p value < 0.1 for interaction was considered suggestive of heterogeneity. SAS version 9.4 (SAS Institute, Cary, NC, USA) and R version 4.4.3 (R Foundation for Statistical Computing, Vienna, Austria) were used for statistical analyses.

## Results

### Study population

**Fig. 1:**
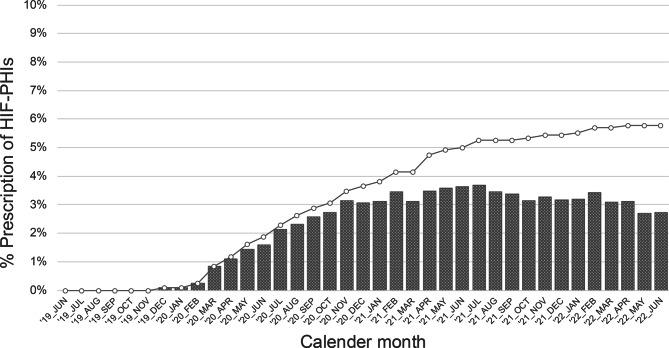
Trends in the proportion of patients prescribed HIF-PHIs during the study period. The bars and lines represent the monthly HIF-PHI prescription proportions and the overall proportion of patients who were ever prescribed an HIF-PHI, respectively. HIF-PHI, hypoxia-inducible factor prolyl hydroxylase inhibitor

During J-DOOPS 7, up to 3.7% of the patients were prescribed HIF-PHIs within 1 month, and a total of 5.8% of the patients received these drugs at least once during the entire period (Fig. 1). When the target was restricted to patients who had received ESA therapy, 3.9% of those were prescribed an HIF-PHI at least once.

**Fig. 2:**
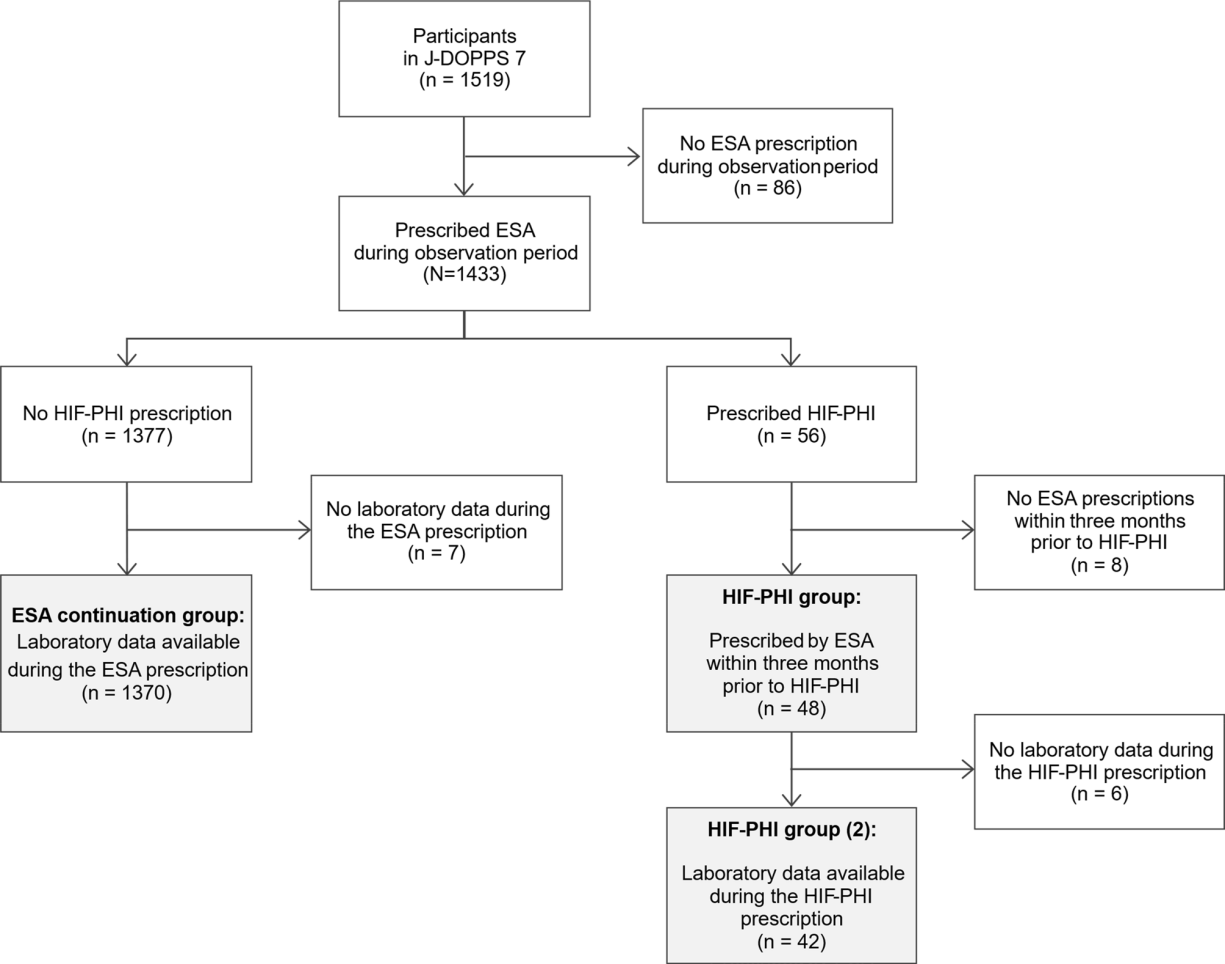
Patient flow. The HIF-PHI group and ESA continuation group comprised Sub-study 1, while HIF-PHI group (2) comprised Sub-study 2. ESA, erythropoiesis-stimulating agent; HIF-PHI, hypoxia-inducible factor prolyl hydroxylase inhibitor

Figure 2 illustrates the patient flow in this study. The analysis population for Sub-study 1 comprised 48 patients who switched from ESA therapy to HIF-PHIs (HIF-PHI group) and 1370 patients who remained on ESA therapy (ESA continuation group). Among the 48 patients in the HIF-PHI group, 32 received roxadustat, 14 received daprodustat, 1 received enarodustat, and the remaining 1 received vadadustat. The analysis population for Sub-study 2 comprised 42 patients (HIF-PHI group (2)); 28 received roxadustat, 12 received daprodustat, 1 received enarodustat, and the remaining 1 received vadadustat.

### Sub-study 1: characteristics of patients who switched to HIF-PHIs

Table 1 summarizes the patient characteristics of the HIF-PHI group and the ESA continuation group. No significant differences were found for age, sex, dialysis history, or causes of end-stage kidney disease between the groups. In the HIF-PHI group, levels of hemoglobin (10.0 vs. 10.7 g/dL), albumin (3.3 vs. 3.6 g/dL), total cholesterol (142 vs. 156 mg/dL), and non–high-density lipoprotein cholesterol (91 vs. 106 mg/dL) were significantly lower, while the proportion of patients prescribed iron agents (52% vs. 30%), ESA dosage (10422 vs. 5203 IU/week), and ERI (18.4 vs. 8.5 IU/kg/week/g/dL) were significantly higher compared with the ESA continuation group. Inflammatory markers showed no significant differences in C-reactive protein levels or neutrophil-to-lymphocyte ratio between the groups, whereas the platelet-to-lymphocyte ratio was significantly lower in the HIF-PHI group (141 vs. 183). With respect to iron-related treatment, the proportion of patients receiving iron agents was higher in the HIF-PHI group (52% vs. 30%), predominantly due to more frequent use of intravenous iron. Although the mean monthly dose of intravenous iron was lower in the HIF-PHI group (205.0 vs. 274.7 mg per month), the total iron exposure per month, calculated as the sum of intravenous iron, oral iron, and iron derived from red blood cell transfusions, was comparable between the two groups.

**Table 1:** Patients’ baseline characteristics

		HIF-PHI group ^1)^ *n* = 48				ESA continuation group ^2)^ *n* = 1370			SMD	*p*-value^3)^
		mean/ median/%	SD/IQR/n	missing		mean/ median/%	SD/IQR/n	missing		
Age [years]		69.1	10.5	0		66.4	12	0	0.24	0.09
Sex (male)		56%	27	0		67%	918	0	0.22	0.12
Body mass index [kg/m^2^]		21.8	3.2	9		22.6	4.3	80	0.21	0.13
Dialysis vintage [years]		6.4	6.3	0		7.4	8.1	0	0.14	0.27
The primary cause of dialysis										
	Diabetes mellitus	36%	17	1		38%	485	109	0.05	0.30
	Glomerulonephritis	40%	19			26%	325		0.32	
	Interstitial nephritis/pyelonephritis	0%	0			1%	11		0.13	
	Cyst/genetic diseases	4%	2			6%	71		0.06	
	Hypertension/Macrovascular diseases	2%	1			5%	64		0.16	
	Other than the above	17%	8			24%	305		0.18	
*Laboratory*										
Blood hemoglobin [g/dL]		10	1.3	0		10.7	1.3	9	0.54	0.001
Serum ferritin [ng/mL]^4)^		101	60.0–258	1		99	48.4–194	84	−	0.62
Transferrin saturation [%]		24.3	13.8	1		25.5	11.1	86	0.10	0.54
Serum iron [μg/dL]		52.1	35.9	1		60	28.4	21	0.24	0.14
Total iron binding capacity [μg/dL]		245	64.3	2		244	48.6	187	0.01	0.93
Serum albumin [g/dL]		3.3	0.4	0		3.6	0.4	10	0.57	0.0004
Total cholesterol [mg/dL]^5)^		142	34.1	12		156	34.1	425	0.41	0.02
HDL cholesterol [mg/dL]^5)^		49.6	16.1	5		49.2	15.7	456	0.03	0.87
Non-HDL cholesterol [mg/dL]^5)^		91	28.3	14		106	32.8	641	0.49	0.005
C-reactive protein [mg/dL]^4)^		0.2	0.05–1.22	4		0.13	0.06–0.43	144	−	0.52
Neutrophil-to-lymphocyte ratio^5)^		4.4	2.3	20		4.0	2.2	648	0.17	0.39
Platelet-to-lymphocyte ratio^5)^		141	73.3	21		183	95	754	0.49	0.008
*Medication*										
Prescription of ESAs		100%	48	0		100%	1370	0	−	
Type of ESAs										
	Darbepoetin alfa	79%	38	0		59%	809	0	0.45	0.04
	Epoetin alfa/epoetin kappa	13%	6			22%	301		0.25	
	Epoetin beta	2%	1			8%	115		0.29	
	Epoetin beta pegol	6%	3			11%	145		0.16	
Dose of ESA [IU/week]		10422	8079	0		5203	4446	4	0.80	<0.0001
ERI [IU/kg/week/g/dL]^6)^		18.4	13.5	8		8.5	8.4	52	0.87	<0.0001
Prescription of iron drugs										
	Intravenous	48%	23	0		25%	336	0	0.50	0.004
	Oral	4%	2			5%	74		0.06	
	Intravenous and Oral	0%	0			0.3%	4		0.08	
Dose of intravenous iron [mg/month] ^7)^		205.0	111.5	0		274.7	134.4	0	0.56	0.008
Total iron exposure [mg/month] ^7)8)^		181.8	212.5	2		177.8	262.4	8	0.02	0.90

Continuous variables are summarized as the mean and SD or the median and IQR. Categorical variables are summarized as proportions (%) and frequencies

1) Values were retrieved from the latest measurements before switching to HIF-PHIs.

2) Values were retrieved from the earliest measurements taken during ESA therapy in the observation period.

3) Welch’s t-test, Mann–Whitney U-test, or chi-square test.

4) Median and interquartile range.

5) Measured at baseline only.

6) ERI = amount of ESA [IU/week]/blood hemoglobin [g/dL]/body weight post-dialysis [kg].

7) Average values over the previous 6 months.

8) Calculated as intravenous iron (mg) + oral iron (mg) × 0.1 + number of transfused red blood cell units × 100 mg per unit (200 mL).

HIF-PHI, hypoxia-inducible factor prolyl hydroxylase inhibitor; ESA, erythropoiesis-stimulating agent; SMD, standardized mean difference; HDL, high-density lipoprotein; ERI, erythropoietin resistance index; SD, standard deviation; IQR, interquartile range

**Fig. 3:**
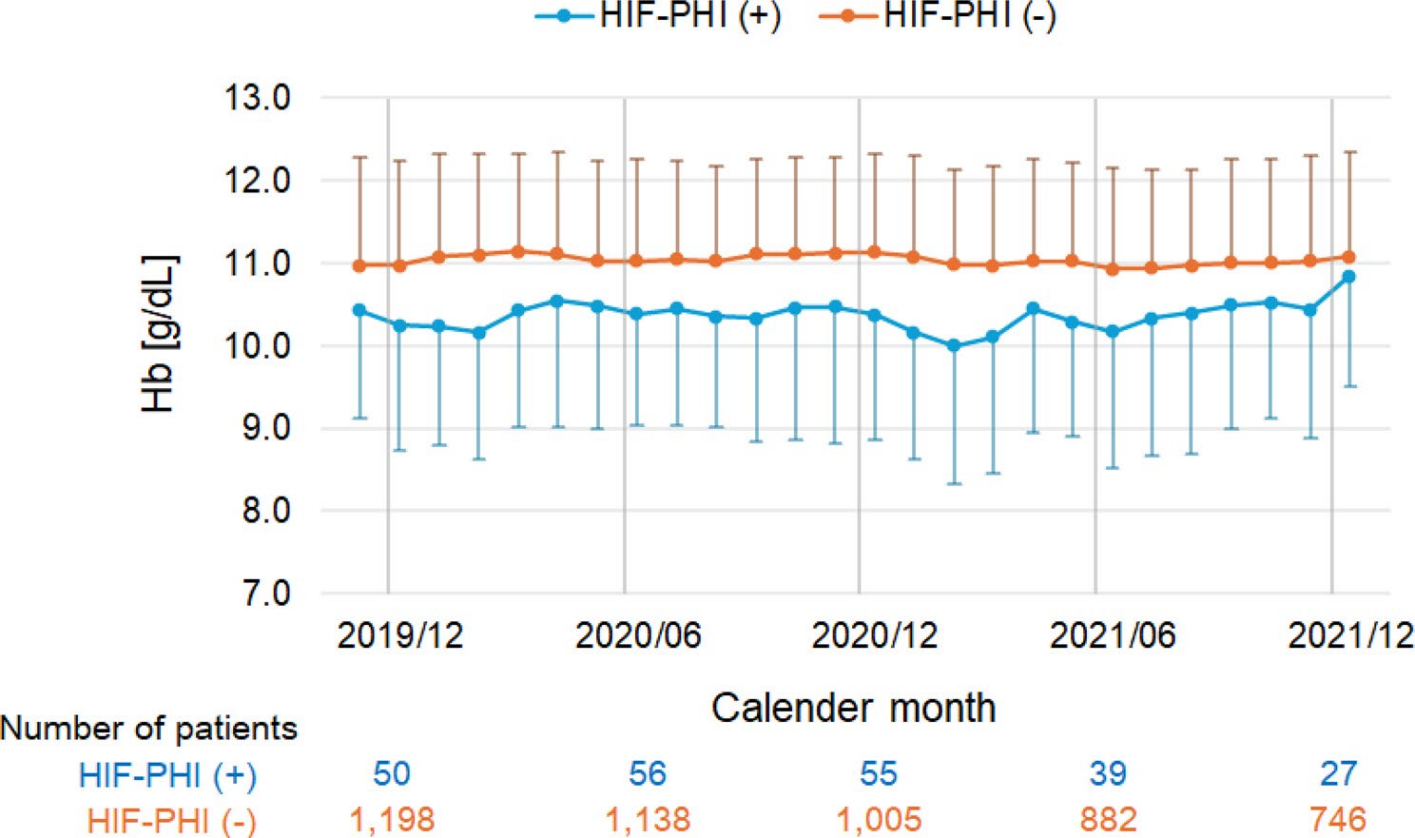
Changes in blood hemoglobin values for the HIF-PHI group and the ESA continuation group [HIF-PHI (+) and HIF-PHI (−), respectively] throughout the study period. The dots and bars represent means and standard deviations, respectively. ESA, erythropoiesis-stimulating agent; HIF-PHI, hypoxia-inducible factor prolyl hydroxylase inhibitor

Changes in Hb in the two groups over the observation period are shown in Fig. 3. The average Hb values in the HIF-PHI group remained lower than those in the ESA continuation group. Regarding the status of iron metabolism in the HIF-PHI group, ferritin remained higher than that in the ESA continuation group, and TIBC showed an upward trend (Supplementary Figure 1). TSAT and serum iron levels exhibited similar, flat transitions in both groups.

### Sub-study 2: changes in anemia status after switching to HIF-PHIs

**Fig. 4:**
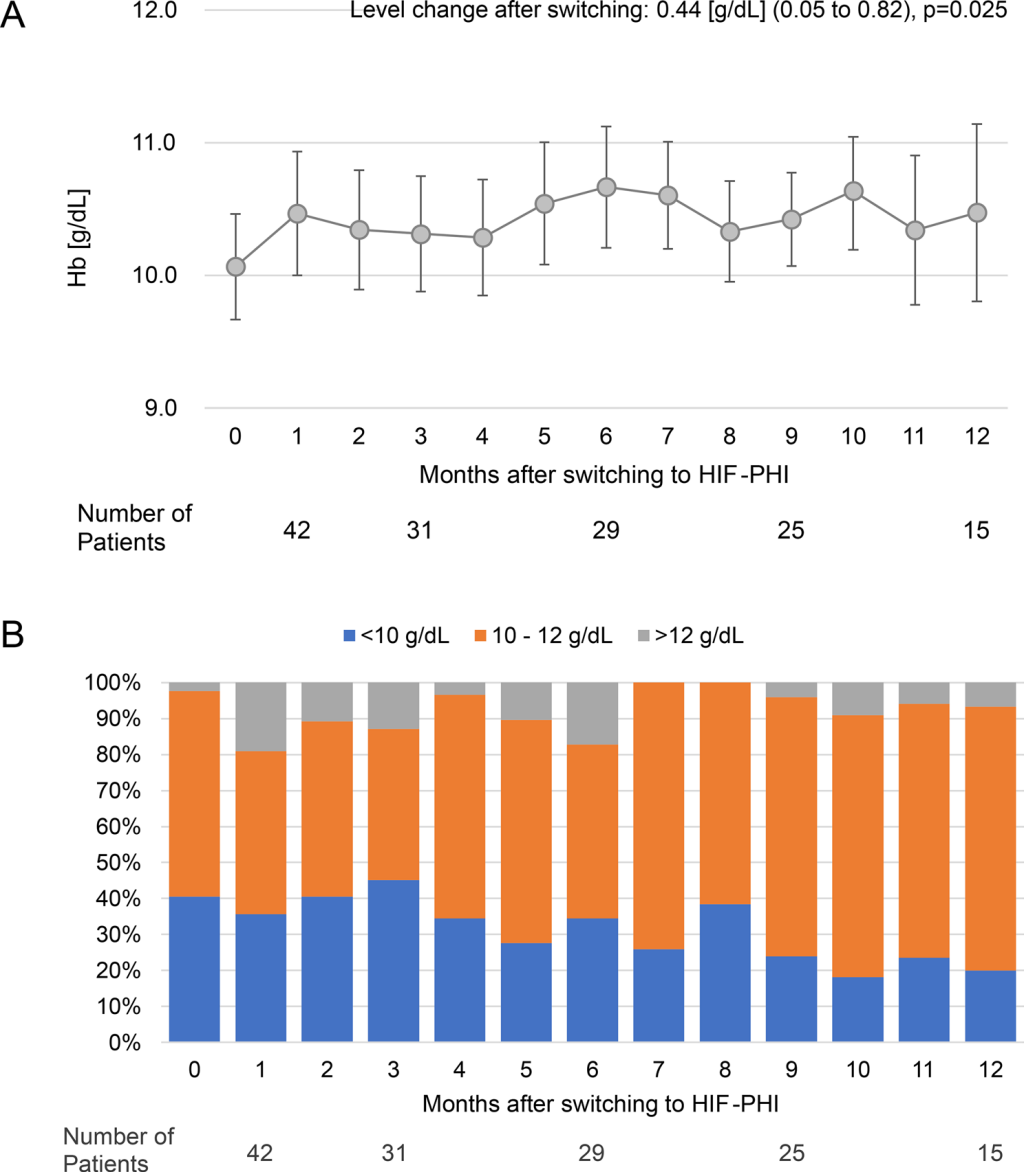
Changes in blood hemoglobin values after switching from ESAs to HIF-PHIs. (**A**): point estimates (dots) and 95% CIs (bars) are shown. Month “0” indicates the most recent measurements taken before the switch. The average change before and after switching to HIF-PHIs, derived by interrupted time series analysis, is noted at the top of the panel. (**B**): categorical presentation of hemoglobin values. CI, confidence interval; ESA, erythropoiesis-stimulating agent; HIF-PHI, hypoxia-inducible factor prolyl hydroxylase inhibitor

Figure 4 shows the changes in Hb values following the switch from ESA therapy to HIF-PHIs. Hemoglobin increased immediately after the switch, and the proportion of patients with Hb values < 10 g/dL decreased over time. When comparing the average values from 6 months before the switch and 12 months after, hemoglobin values were an average of 0.44 g/dL (95% CI: 0.05–0.82; *p* = 0.025) higher after switching. Regarding iron metabolism (Supplementary Figure 1), TSAT and ferritin did not change significantly after the switch. In contrast, serum iron and TIBC values increased starting 1 month after the switch, with significant differences noted between the average values for 6 months before the switch and 12 months following the switch.

**Fig. 5:**
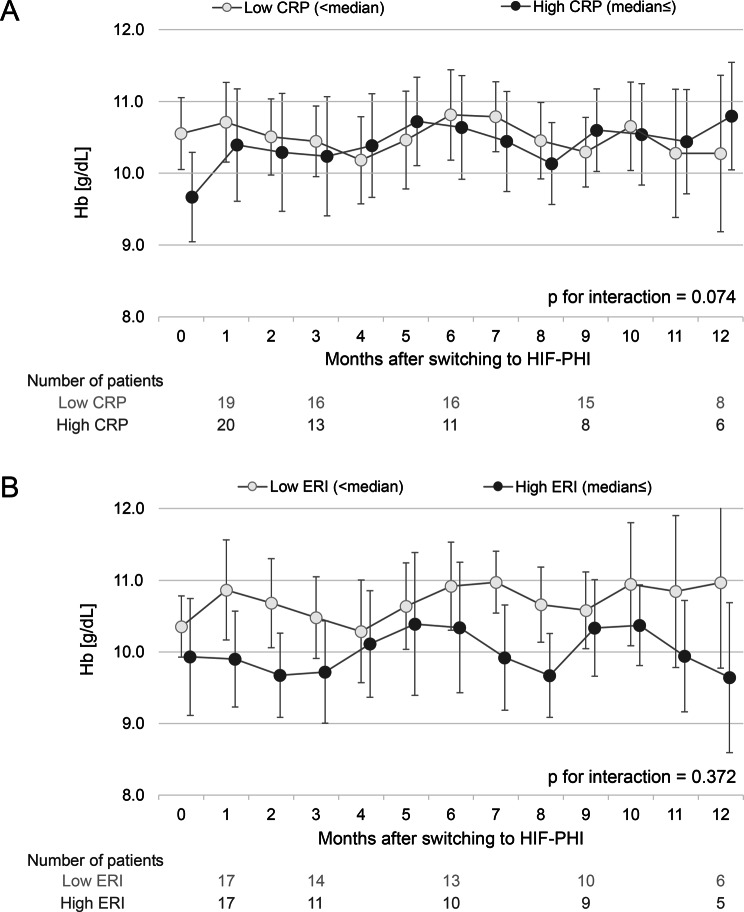
Interactions between the change in blood hemoglobin after switching from ESAs to HIF-PHIs and CRP (**A**) or ERI (**B**). CRP and ERI were divided into two groups on the basis of the median values. Point estimates (dots) and 95% CIs (bars) are presented. Month “0” represents the most recent measurements taken before the switch. CI, confidence interval; CRP, C-reactive protein; ERI, erythropoietin resistance index; ESA, erythropoiesis-stimulating agent; HIF-PHI, hypoxia-inducible factor prolyl hydroxylase inhibitor

When examining the interaction between Hb changes before and after the switch and CRP (Fig. 5A), Hb values tended to increase after switching in the group with high CRP levels relative to the group with low CRP levels (p for interaction = 0.074). In contrast, no differences in Hb changes were observed between the high ERI group and the low ERI group (Fig. 5B; p for interaction = 0.372).

## Discussion

HIF-PHIs have emerged as a novel therapeutic option to replace ESAs and were first approved in China in December 2018 for DD-CKD patients [19]. Clinically, HIF-PHI use in dialysis patients began in approximately July 2019 in both China and Japan. In the European Union, roxadustat received regulatory approval in 2021, although its uptake in clinical practice has remained limited. In the USA, the Food and Drug Administration approved daprodustat in February 2023 and vadadustat in March 2024 for the treatment of anemia in DD-CKD patients. While these approvals represent a major advancement in the global availability of HIF-PHIs, real-world evidence regarding their clinical efficacy and safety remains limited, and further clinical data are needed to inform optimal patient selection and therapeutic strategies.

This study aimed to clarify the clinical characteristics and outcomes of maintenance HD patients newly initiated on HIF-PHIs, using real-world data from phase 7 of J-DOPPS (July 2019–September 2022). Specifically, we compared patients who switched from ESAs to HIF-PHIs with those who continued ESA therapy, and evaluated Hb responses stratified by ESA resistance and inflammation status.

Among 1433 patients who were prescribed ESAs during the observation period, only 56 patients (3.9%) were switched to HIF-PHIs. The low proportion of patients switched to HIF-PHIs in this cohort likely reflects several real-world factors. First, during the early phase of HIF-PHI availability in Japan, concerns regarding long-term safety, particularly thromboembolic risk, may have limited their widespread adoption, as highlighted by expert recommendations such as those by the Asian Pacific Society of Nephrology [20]. Second, because the mean baseline hemoglobin level in the ESA continuation group was relatively well controlled (10.7 g/dL) with moderate ESA dosing, clinicians may have perceived limited clinical necessity to change established anemia management strategies. Finally, limited clinical experience and familiarity with HIF-PHIs during the early post-approval period may also have contributed to a cautious prescribing approach. Compared with the ESA continuation group, respectively, patients in the HIF-PHI group exhibited poorer nutritional status (albumin: 3.3 vs. 3.6 g/dL; total cholesterol: 142 vs. 156 mg/dL) and, despite receiving significantly higher ESA doses (10422 vs. 5203 IU/week) and higher iron prescription rates (52% vs. 30%), presented with lower Hb levels (10.0 vs. 10.7 g/dL) and significantly greater ERIs (18.4 vs. 8.5 IU/kg/week/g/dL), suggesting selection of more treatment-refractory cases for HIF-PHI initiation.

Following the switch to an HIF-PHI, a significant increase in Hb was observed at 1 month (0.44 [95% CI: 0.05–0.82] g/dL; *p* = 0.025). Serum iron and TIBC also increased significantly, although no clear changes were noted in TSAT or ferritin levels.

HIF-PHIs exert pleiotropic effects beyond erythropoiesis via HIF activation, including cholesterol reduction, improved iron metabolism, and potential anti-inflammatory actions [16]. As stated, patients switched from ESA to HIF-PHI were selected among those with anemia resistant to treatment and were characterized by high iron prescription rates and ERIs. This suggests that these patients were chosen with the expectation of augmented treatment response due to improved inflammation-related abnormalities in iron metabolism.

Stratified analysis by inflammation and ESA resistance revealed a trend toward a greater Hb response in patients with high CRP levels compared with those with low CRP (p for interaction = 0.074), suggesting that HIF-PHIs may remain effective even in the presence of inflammation. In contrast, no significant difference in Hb response was observed between the high- and low-ERI subgroups (p for interaction = 0.372). Importantly, as this was a real-world observational study, confounding by indication represents a major limitation in the interpretation of our findings. Patients who were switched to HIF-PHIs showed markedly higher ESA resistance, as reflected by a substantially higher ERI and lower baseline hemoglobin levels. Although ESA hyporesponsiveness is commonly attributed to inflammation-associated functional iron deficiency, patients in the HIF-PHI group exhibited lower serum albumin and cholesterol levels, while C-reactive protein and serum ferritin levels were not markedly elevated. These findings suggest that the HIF-PHI group may have been characterized more by malnutrition-related ESA hyporesponsiveness rather than a predominantly inflammatory phenotype. In such cases, where ESA resistance is driven by insufficient nutritional substrates rather than hepcidin-mediated iron sequestration, improvements in serum iron parameters and total iron-binding capacity may not necessarily translate into meaningful correction of anemia. Therefore, the observed treatment responses should be interpreted with caution, taking into account the underlying clinical context of ESA hyporesponsiveness in this population.

HIF-1, induced by HIF-PHIs, promotes the expression of transferrin and its receptor, which are involved in iron transport and cellular uptake. Furthermore, by suppressing the expression of hepcidin, which causes functional iron deficiency, HIF-1 is believed to induce efficient hematopoiesis [21]. However, the accelerated hematopoiesis induced by HIF-PHIs, with the rapid improvement in iron metabolism, can increase the body’s iron demand and the possibility of relative iron deficiency. This, in turn, raises concerns about a potential increase in the risk of thrombotic events. Basic research has reported that transferrin, which has increased expression via hypoxia response elements that are activated by iron deficiency, binds to prothrombin and factor XII, which promotes coagulation [22, 23]. Clinically, a meta-analysis of roxadustat in NDD-CKD patients demonstrated a significantly higher risk of deep vein thrombosis compared with placebo (relative risk: 3.80; 95% CI: 1.50–9.64) [24]. Similarly, in the ASCEND-ND trial, NDD-CKD patients treated with daprodustat had a higher incidence of vascular access thrombosis compared with those treated with ESA [25]. Furthermore, while reports are inconsistent, some phase 3 trials in dialysis patients also suggest a higher incidence of arteriovenous shunt thrombosis in the HIF-PHI group compared with the ESA group [26, 27].

This study and other reports on both NDD-CKD [25, 28] and DD-CKD [26, 27] patients have observed increases in transferrin and TIBC following HIF-PHI treatment, highlighting the need for further investigation into the relationship between these changes and thrombotic complications.

Our study also found that the HIF-PHI group had a significantly higher rate of iron supplementation than the ESA group, which may reflect a clinical strategy to prevent the development of functional iron deficiency. In contrast, previous reports have indicated a decrease in ferritin levels after HIF-PHI administration [25–28]. However, in the present study, a clear decrease in ferritin levels was not observed 1 month after switching from ESA to HIF-PHI. Prior studies on daprodustat have also reported that the magnitude of decrease in ferritin levels was almost comparable between the daprodustat and ESA groups [29–32]. This suggests that the impact on ferritin levels may be influenced by individual iron supplementation status, including the presence or absence of iron administration and the dosage.

Our findings also suggest that HIF-PHIs may be more effective in patients with vs. without inflammation. Indeed, previous studies have reported that HIF-PHIs effectively correct and maintain Hb levels even in inflammatory states with elevated CRP values, both in NDD-CKD [28, 33] and DD-CKD [27, 32, 34] patients.

Regarding ESA hyporesponsiveness, our study did not detect a significant difference in Hb response between the high- and low-ERI subgroups. A previous report from Japan found that, as ERI increased, the required dose escalation was greater for darbepoetin alfa than for daprodustat, supporting the efficacy of HIF-PHIs in patients with high ESA resistance [29]. However, the ASCEND-TD trial, which included primarily White patients, reported that daprodustat was not superior to ESAs in ESA-hyporesponsive patients, indicating that efficacy may vary depending on ethnicity or clinical background [30].

In this context, our findings are also informative when interpreted in light of the recently published 2026 KDIGO Clinical Practice Guideline for Anemia in CKD, which positions HIF-PHIs as second-line agents following ESAs. In the present study, HIF-PHIs were predominantly prescribed to patients with ESA-hyporesponsive or refractory anemia, reflecting a real-world treatment strategy that is consistent with the KDIGO framework. Our results suggest that, when used in this selected population, HIF-PHIs can improve hemoglobin levels and iron metabolism, even in patients with markers of inflammation, while underscoring the importance of careful patient selection and monitoring. These real-world data provide complementary evidence to support the guideline-recommended role of HIF-PHIs as a second-line therapeutic option rather than a universal replacement for ESAs [35].

In our study, the median ERI in the HIF-PHI group was 14.6 IU/kg/week/g/dL—markedly high compared with previously reported ERI values in Japanese dialysis patients [12, 29]. Thus, direct comparison with low-ERI patients was limited, representing a key area for future investigation.

This study provides important real-world data from a prospective observational cohort, offering valuable insight into the clinical impact of HIF-PHIs. However, there are several limitations in the present study. First, HIF-PHIs had only recently become available during the study period and had not yet achieved widespread use in routine clinical practice; therefore, the number of patients who switched from ESA therapy was limited. Second, these agents were initially introduced with the expectation of being effective in treatment-resistant anemia, leading clinicians to preferentially prescribe them to CKD patients with ESA-hyporesponsive or refractory anemia. This selection bias represents another key limitation of the study. Third, these data were obtained during the early period of HIF-PHI introduction in Japan (2019–2022). Since prescribing patterns may have changed since then, the findings may not necessarily be generalizable to all current dialysis patients. In addition, anemia management practices in Japan differ from those in other regions. In the present study, only 30% of patients in the ESA continuation group were prescribed iron agents, reflecting the relatively low rate of iron use among hemodialysis patients in Japan. Previous DOPPS data have shown that iron utilization rates in Japan are substantially lower than those reported in other regions, such as the United States, where more than 80% of hemodialysis patients receive iron therapy. Therefore, caution is warranted when extrapolating our findings to settings with different anemia management strategies and higher background use of iron supplementation. Fourth, this study focused on efficacy outcomes such as Hb levels and iron metabolism, and did not assess long-term safety concerns associated with HIF-PHIs, such as thromboembolic events. Therefore, further accumulation of real-world data is needed to evaluate the effectiveness of HIF-PHIs in a broader population of CKD patients, including those with more general anemia responsiveness.

## Conclusion

In this real-world analysis, patients on hemodialysis who switched from ESA to HIF-PHI therapy tended to have high ESA doses and elevated ERIs, with a greater need for iron supplementation. One month after switching, a significant increase in Hb was observed, and even patients with elevated CRP levels demonstrated a favorable hematologic response. Further studies involving larger patient populations and long-term safety evaluations are warranted.

## Electronic supplementary material

Below is the link to the electronic supplementary material.

Supplementary Material 1

## Data Availability

The data that support the findings of this study are available from Arbor Research Collaborative for Health but restrictions apply to the availability of these data, which were used under license for the current study, and so are not publicly available. Data are however available from the authors upon reasonable request and with permission of Arbor Research Collaborative for Health.

## Abbreviations

CI: Confidence interval
CKD: Chronic kidney disease
CRP: C-reactive protein
DD: Dialysis-dependent
DOPPS: Dialysis Outcomes and Practice Patterns Study
ERI: Erythropoietin resistance index
ESA: Erythropoiesis-stimulating agent
GEE: Generalized estimation equation
Hb: Hemoglobin
HD: Hemodialysis
HIF: Hypoxia-inducible factor
HIF-PHI: Hypoxia-inducible factor prolyl hydroxylase inhibitor
IU: International unit
J-DOPPS: Japanese Dialysis Outcomes and Practice Patterns Study
KDIGO: Kidney Disease: Improving Global Outcomes
NDD: Non–dialysis-dependent
TIBC: Total iron-binding capacity
TSAT: Transferrin saturation
