# Supplementary material for: Real-world use of HIF-PH inhibitors in the Japan dialysis outcomes and practice patterns study (J-DOPPS, 2019–2022)
Source: BMC Nephrol. 2026 Feb 19;27:250. doi: 10.1186/s12882-026-04831-2 (PMC13097766; doi:10.1186/s12882-026-04831-2)
Supplement: Supplementary file 1 — Supplementary Material 1 [file 12882_2026_4831_MOESM1_ESM.docx]

**Real-World Use of HIF-PH Inhibitors in the Japan Dialysis and Practice Patterns Study (J-DOPPS, 2019-2022)**

Masahiro Eriguchi^1^, Kazuhiko Tsuruya^1^, Hisashi Noma^2^, Yoshihiro Onishi^3,4^, Masami Inuzuka^5^, Kenji Harada^5^, Masaomi Nangaku^6^,

1. Department of Nephrology, Nara Medical University, Kashihara, Japan

2. Department of Data Science, The Institute of Statistical Mathematics, Tachikawa, Japan.

3. Institute for Health Outcomes and Process Evaluation Research (iHope International), Kyoto, Japan

4. Patient Driven Academic League (PeDAL), Kyoto, Japan

5. Medical Affairs Department, Kyowa Kirin Co., Ltd., Tokyo, Japan.

6. Division of Nephrology and Endocrinology, The University of Tokyo Graduate School of Medicine, Tokyo, Japan.

Corresponding Author, Masahiro Eriguchi, MD, PhD

Department of Nephrology, Nara Medical University

840 Shijo–cho, Kashihara, Nara, 634-8521, Japan

Tel: +81–744–29–8865 Fax: +81–744–23–9913

e–mail: meriguci@gmail.com

**Supplementary Figure 1.** Changes in iron metabolism markers in the HIF-PHI group and the ESA continuation group during the study period.


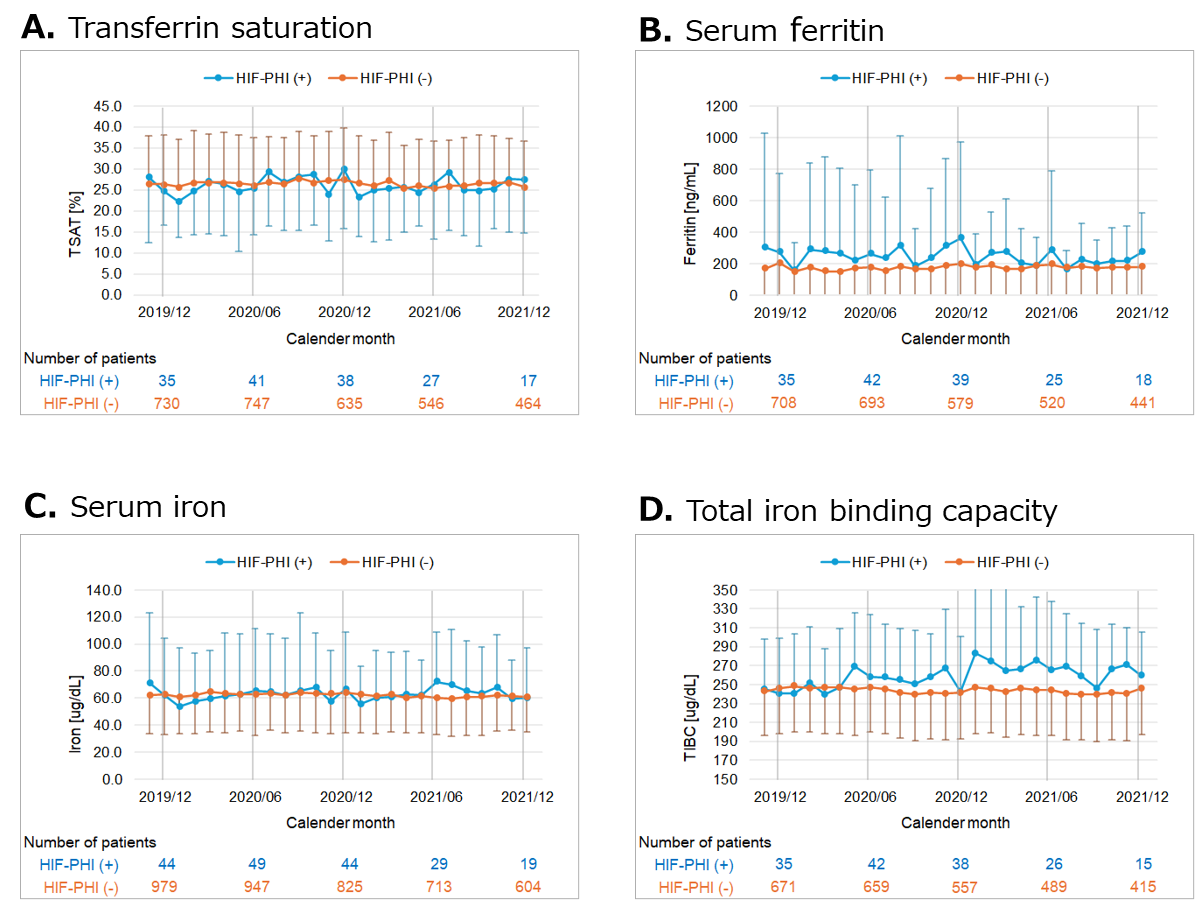


HIF-PHI (+); the HIF-PHI group, and HIF-PHI (−); the ESA continuation group.

The dots and bars represent means and standard deviations, respectively.

**Supplementary Figure 2.** Changes in iron metabolism markers after switching from ESA to HIF-PHI.


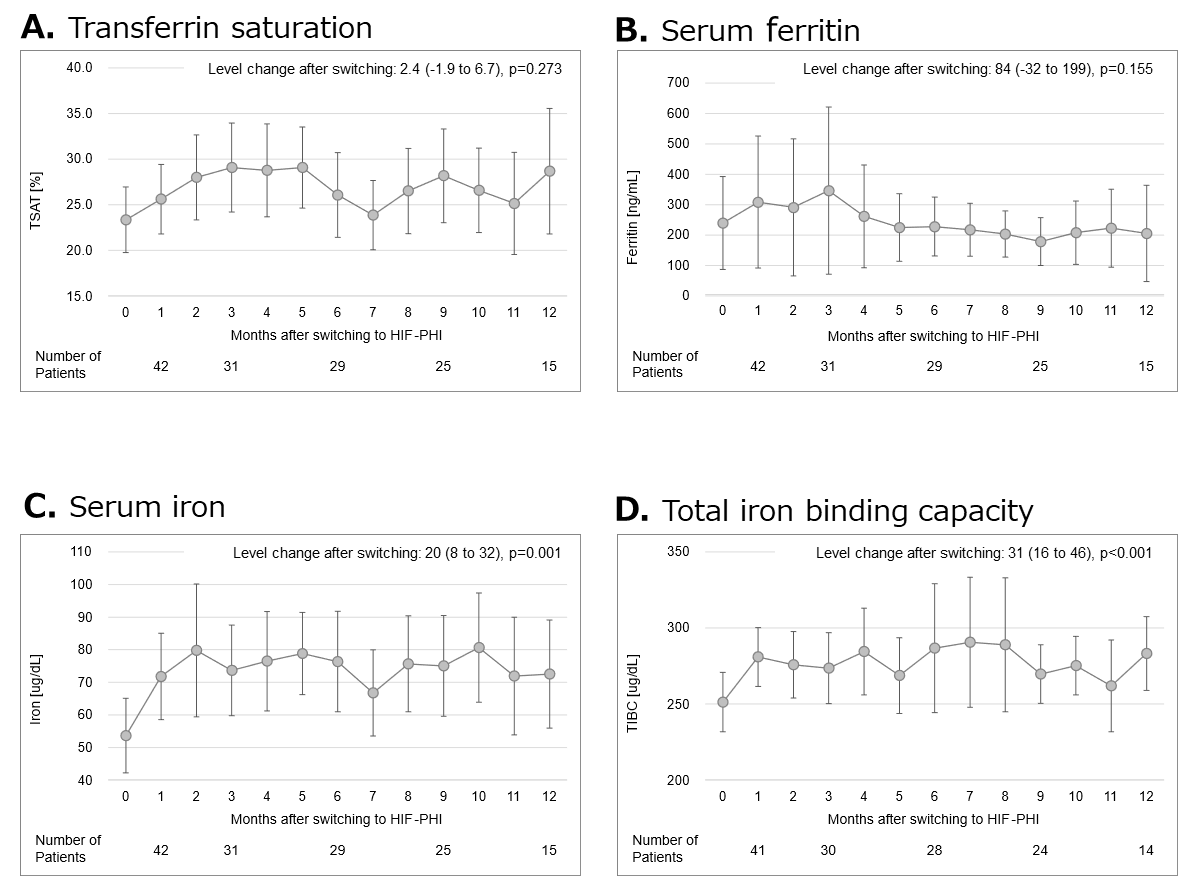


Point estimates (dots) and 95% confidence intervals (bars) are shown. The month ‘0’ represents the most recent measurements taken before the switch. The average change before and after the switch to HIF-PHIs, derived by interrupted time series analysis, is noted at the top of each panel.
